# Peer Support Needs and Preferences for Digital Peer Navigation among Adolescent and Young Adults with Cancer: A Canadian Cross-Sectional Survey

**DOI:** 10.3390/curroncol29020099

**Published:** 2022-02-16

**Authors:** Jacqueline L. Bender, Natasha Puri, Sarah Salih, Norma M. D’Agostino, Argerie Tsimicalis, A. Fuchsia Howard, Sheila N. Garland, Karine Chalifour, Emily K. Drake, Anthony Marrato, Nikki L. McKean, Abha A. Gupta

**Affiliations:** 1 Cancer Rehabilitation and Survivorship, Princess Margaret Cancer Centre, University Health Network, Toronto, ON M5G 2C4, Canada; npuri@sgu.edu (N.P.); sarah.salih@uhnresearch.ca (S.S.); anthony@marrato.com (A.M.); nikki@nikkileighmckean.com (N.L.M.); 2 Department of Supportive Care, Princess Margaret Cancer Centre, University Health Network, Toronto, ON M5G 2C1, Canada; norma.dagostino@uhn.ca (N.M.D.); abha.gupta@uhn.ca (A.A.G.); 3 Dalla Lana School of Public Health, University of Toronto, Toronto, ON M5T 3M7, Canada; 4 Institute of Health Policy, Management and Evaluation, University of Toronto, Toronto, ON M5T 3M7, Canada; 5 Ingram School of Nursing, McGill University, Montreal, QC H3A 2M7, Canada; argerie.tsimicalis@mcgill.ca; 6 School of Nursing, The University of British Columbia, Vancouver, BC V6T 2B5, Canada; Fuchsia.Howard@ubc.ca; 7 Department of Psychology, Memorial University, St. John’s, NL A1C 3X9, Canada; sheila.garland@mun.ca; 8 Young Adult Cancer Canada, St. John’s, NL A1B 3K3, Canada; karine@youngadultcancer.ca; 9 Faculty of Health, Dalhousie University, Halifax, NS B3H 4R2, Canada; emily.drake@dal.ca; 10 Adolescent and Young Adult Cancer Program, Princess Margaret Cancer Centre, University Health Network, Toronto, ON M5G 2C1, Canada

**Keywords:** adolescents and young adults, cancer, peer support, navigation, digital health

## Abstract

Adolescents and young adults (AYA) with cancer desire peer support and require support programs that address their unique needs. This study investigated the need for, and barriers to, peer support and preferences for digital peer navigation among AYA. A cross-sectional survey was administered to AYA, diagnosed with cancer between the ages of 15–39, at a cancer center and through social media. Descriptive summary statistics were calculated. Participants (*n* = 436) were on average 31.2 years (SD = 6.3), 3.3 years since-diagnosis (SD = 3.8), and 65% (*n* = 218) were women. Over three-quaters (*n* = 291, 76.6%) desired peer support from cancer peers, but 41.4% (*n* = 157) had not accessed peer support. Main access barriers were: Inconvenience of in-person support groups (*n* = 284, 76.1%), finding AYA with whom they could relate (*n* = 268, 72.4%), and finding AYA-specific support programs (*n* = 261, 70.4%). Eighty-two percent (*n* = 310) desired support from a peer navigator through a digital app, and 63% (*n* = 231) were interested in being a peer navigator. Participants indicated a greater need for emotional (*n* = 329, 90.1%) and informational support (*n* = 326, 89.1%) than companionship (*n* = 284, 78.0%) or practical support (*n* = 269, 73.6%) from a peer navigator. Foremost peer matching characteristics were cancer-type (*n* = 329, 88.4%), specific concerns (*n* = 317, 86.1%), and age-at-diagnosis (*n* = 316, 86.1%). A digital peer navigation program was desired by over 80% of a large Canadian sample of AYA and could potentially overcome the barriers AYA experience in accessing peer support. The design of a peer navigation program for AYA should consider the matching characteristics and multidimensional support needs of AYA.

## 1. Introduction

Adolescents and young adults (AYA; defined as individuals diagnosed with cancer between the ages of 15 and 39) comprise a distinct cancer population with unique needs [1]. Due to their age and life stage at diagnosis, AYA are confronted with a multitude of physical, psychosocial, and socioeconomic impacts of cancer that are disproportionately greater than in older adults [1]. AYA report higher levels of distress, more severe side effects, interrupted education, careers, and relationships, disconnection from peers, and financial hardship [2,3,4,5,6]. Many AYA lack the life experience and skills to cope with these significant challenges [7], yet healthcare systems provide inadequate or inaccessible support [8,9,10], resulting in a high burden of unmet needs [9,10,11,12]. 

To overcome these significant challenges, support programs that connect AYA with same-age cancer peers, as well as provide practical knowledge and skills to navigate the healthcare system are needed [13,14,15]. In a Canadian hospital-based survey, 56% of 127 AYA that were surveyed indicated a desire for peer support from other AYA [16]. In a Canadian qualitative study, AYA recommended a buddy system linking patients to peer mentors to navigate the healthcare system [14]. Support from a cancer peer provides unique reassurance and practical information that can reduce distress and enhance coping [17,18,19,20]. However, AYA report limited opportunities to interact with other AYA and encounter significant challenges in forming new social relationships [11].

Patient navigation is a well-documented approach that can overcome gaps in access to care and support [21,22,23,24,25,26]. Peer navigation, a type of patient navigation, enlists trained cancer survivors as navigators [27]. Digital technologies could enhance access to peer navigation and are preferred by AYA [28]. However, few studies have investigated navigation among AYA, and even less have explored the use of digital technologies to facilitate navigation [22]. One qualitative study of the navigation preferences of American AYA, found that all developmental age groups (e.g., 15–18, 19–25, 26–39) were interested in navigation, with most preferring an initial face-to-face interaction followed by a technology-mediated interactions (e.g., by telephone, text or email) [15]. One randomized controlled trial compared an automated text-messaging support intervention versus telephone-based peer navigation (from same-aged non-cancer peers) to usual care among a sample of American AYA [29]. The text messaging group had higher survivorship care knowledge, while the peer navigation group had higher survivorship care self-efficacy compared to usual care [29].

Research is needed to better understand the peer support needs of Canadian AYA and their interest in peer navigation. While the US data are helpful, they cannot be directly extrapolated to the Canadian context, given the different healthcare and social systems. Therefore, this study examined the need for, and barriers to, peer support and preferences for digital peer navigation among a sample of AYA in Canada. Guided by the Canadian Strategy for Patient-Oriented Research (SPOR) [30], AYA were engaged as patient partners and collaborators on the study team. There are many benefits to engaging patients in health research, not limited to improvements in research relevance, patient enrollment and retention, as well as the translation of study findings into clinical practice [31,32]. Moreover, for the present study, engaging with patient partners from the onset was critical to inform the design of a future solution tailored to the self-reported peer support needs and preferences of AYA.

## 2. Materials and Methods

### 2.1. Study Design

A cross-sectional survey design was employed, following the Strengthening the Reporting of Observational Studies in Epidemiology (STROBE) guidelines [33]. Ethics approval was obtained by the University Health Network (UHN) Research Ethics Board.

### 2.2. Setting

The survey was distributed to AYA at the Princess Margaret Cancer Center (PM) in Toronto, Ontario, Canada, and online from September 2018 to April 2019. The PM has an AYA Program that provides specialized support to younger people (less than 40 years old) living with cancer. Patients who participate in the PM AYA Program meet one-on-one with an AYA Clinical Nurse Specialist to identify where additional support is needed. Patients can sign up to receive a monthly e-newsletter that provides program updates and information on upcoming AYA-specific events. The PM AYA Program also supports an 8-week Mindfulness-Based Cognitive Therapy course, a 6-week music therapy group, and monthly virtual peer support drop-in meetings for AYA. All of the resources and events are promoted online through the AYA PM social media (SM) channels.

At PM, AYA were recruited using a weekly report that identifies all patients who are less than 40 years of age attending disease site ambulatory clinics, including those diagnosed with: leukemia, lymphoma, breast cancer, genitourinary cancer, central nervous system cancer, head/neck cancer, sarcoma, gastrointestinal cancer, and gynecological cancer. Patients were approached during routine appointments, informed about the survey, and if agreeable, consented to study participation, and were given a paper copy of the survey. 

To recruit AYA outside of PM, the study team distributed the survey via SM using a targeted digital recruitment strategy, that followed Bender’s privacy-by-design framework for online recruitment [34]. A recruitment notice was posted on team members’ SM channels and on the PM AYA program’s Twitter account, Instagram channel, and Facebook page, with a link to the online survey. The SM recruitment notice contained a concise study description, an image of young adults, a disclaimer regarding the privacy limitations of SM, and the contact information for the study team. The SM recruitment post with the survey link was shared along with relevant hashtags, including #AYACSM (Adolescent and Young Adult Cancer Societal Movement). The study information was also shared by email to members of the PM AYA Program e-newsletter listserv, community partners, and individual contacts. Specific community partners who shared the SM recruitment notice included Young Adult Cancer Canada (YACC) and members of the #AYACSM community. 

To encourage survey completion, participants were offered the opportunity to be entered into a draw to win one of three CAD $100 Amazon gift cards upon completion of the survey. 

### 2.3. Participants

Individuals were eligible to participate if they were diagnosed with cancer between the ages of 15 to 39 years, able to read and speak English, and were receiving treatment for cancer or within 10 years of treatment completion. 

### 2.4. Questionnaire

The survey contained four sections and 57 questions comprising standardized and author-developed measures. It was pilot tested with four AYA before administration. The four survey sections were, “A. Cancer Specific Background Information”, “B. Preferences for Peer Support from other Adolescents and Young Adults (AYA) Diagnosed with Cancer”, “C. Overall Health and Wellbeing”, and “D. Background Information About You”. This manuscript reports on responses to questions in sections A, B and D, including participant demographics and disease characteristics, the need for and barriers to peer support, and interest and preferences for digital peer navigation (Supplementary Materials). 

### 2.5. Statistical Methods

Descriptive analyses were conducted using SPSS version 25. Means and standard deviations were calculated for continuous variables and proportions for categorical variables. Response options for several demographic characteristics, as well as for items querying satisfaction with peer support received from other AYA and importance of training for the peer navigator role were collapsed and are described in the subsequent tables. For barriers to peer support, response options were dichotomized into no problem versus a problem (somewhat of a problem, big problem, not sure). For matching characteristics, response options were dichotomized into not at all important versus important (not sure, somewhat important, important). For the type of support desired from a peer navigator, response options were dichotomized into not at all important versus important (slightly, moderately, important, very important).

## 3. Results

### 3.1. Participant Characteristics

In total, 436 participants completed the survey, of which 217 (49.8%) participants were recruited from PM and 219 (50.2%) through SM. Participants were on average 31 years of age and 3.3 years post diagnosis (Table 1). Most were diagnosed with breast cancer (*n* = 91, 21.7%) and Hodgkin’s Lymphoma (*n* = 47, 11.2%), with fairly equal numbers of participants diagnosed with testicular cancer, leukemia, sarcoma, and thyroid cancer (8–9% each). About two-thirds of the participants identified as women (*n* = 218, 65.1%), heterosexual (*n* = 286, 64.6%), and white (*n* = 212, 63.1%), and had completed at least some university (*n* = 216, 63.1%). Most of the participants lived in urban or suburban settings (*n* = 292, 86.9%) in the province of Ontario (*n* = 274, 81.3%).

### 3.2. Need for and Use of Peer Support

Over three-quarters of the participants (*n* = 291, 76.6%) desired peer support from other AYA since their diagnosis (Table 2). However, 41.4% (*n* = 157) had not accessed peer support. Two-thirds (*n* = 237, 62%) reported that they had discussed peer support with a medical professional. Of these, 50% (*n* = 191) indicated that their medical professional initiated the conversation, while 12% (*n* = 46) indicated that they had initiated the conversation regarding peer support. Slightly more than a third (*n* = 143, 37.3%) received a referral to a peer support program. The most common method of connecting with other AYA was through SM (*n* = 152, 40.1%). 

Participants indicated a greater need for emotional (*n* = 168, 45.2%), informational (*n* = 159, 42.7%), and companionship support (e.g., hang out and perform social things) (*n* = 161, 43.6%) from other AYA, than practical support (*n* = 105, 28.5%). Of the participants who received peer support from other AYA (*n* = 198, 52.2%), 70% (*n* = 139) were satisfied with the support they received.

### 3.3. Barriers to Accessing Peer Support

The top three barriers to accessing peer support from other AYA, reported by more than 70% of the sample, were the inconvenience of in-person support groups (*n* = 285, 76.1%), finding AYA with whom they can relate to (*n* = 268, 72.4%), and finding AYA- specific support programs (*n* = 261, *n* = 70.4%) (Table 3). The least commonly endorsed access barriers were related to technical challenges. Less than 8% found SM difficult to use, less than 7% had difficulty accessing a computer or mobile device, and less than 4% had difficulty using a computer or a mobile device. 

### 3.4. Interest and Preferences for Digital Peer Navigation 

Half of the sample (*n* = 186, 48.9%) indicated a desire to connect with a peer navigator who is a cancer survivor and who has received training to help others through the cancer experience (Table 4). Two-thirds indicated a desire to be a peer navigator (*n* = 231, 62.9%) and willingness to attend a peer navigator training program (*n* = 231, 63.1%).

Among those who did not desire a peer navigator, 42.1% (*n* = 195) felt they did not need support and 45.1% (*n* = 88) believed they had adequate support. A smaller proportion (*n* = 21, 10.8%) indicated that they did not like to talk about their problems.

The majority of participants (*n* = 310, 82%) indicated a desire to connect with a peer navigator if they could through a digital app, where they could view the profiles of AYA that match their criteria and select an AYA to talk with.

Participants indicated a greater need for emotional (*n* = 329, 90.1%) and informational support (*n* = 326, 89.1%) from a peer navigator than companionship (*n* = 284, 78.0%) or practical support (*n* = 269, 73.6%). 

### 3.5. Peer Matching Characteristics

Ten matching characteristics were endorsed as important by over 50% of the sample (Table 5). The most important matching characteristics, endorsed by over 70% of the sample, were type of cancer (*n* = 329, 88.4%), specific concerns (*n* = 317, 86.1%), age-at-diagnosis (*n* = 316, 86.1%), treatments received or considering (*n* = 314, 85.1), current age (*n* = 302, 82.1%), stage-of-disease (*n* = 278, 75.7%), and coping style (*n* = 268, 73.0%). The least important matching characteristics were sexual orientation (*n* = 63, 17.2%) and race/ethnicity (*n* = 29, 7.9%). 

## 4. Discussion

It is evident from our study that the majority of AYA with cancer desire peer support from other AYA, as well as specialized support from a peer navigator. The results of this study will inform the development of an AYA-specific digital peer navigation program, which, to date, has not been explored. Although the majority of participants indicated a desire for peer support, AYA rarely asked their healthcare teams about peer support, and rather relied on their healthcare professionals to raise the topic. A similar reluctance to seek support for physical and psychosocial concerns was noted in a national population-based study of AYA [9], with participants citing not wanting to ask for help, being told that it was normal to feel the way they did or embarrassment as reasons for not seeking help [9]. Other research has found that AYA are often uncomfortable discussing non-medical issues with healthcare professionals [15], highlighting the importance of training healthcare professionals to be proactive in raising salient issues and referring AYA to available support resources [14].

Despite the widespread desire for peer support, many were still not able to access it. AYA rated the inconvenience of in-person support groups, the difficulty of finding AYA with whom they could relate, and the lack of AYA-specific support programs as the most important access barriers. AYA require and prefer AYA-specific support programs due to their distinct needs related to their age and life stage [12,13,14] and have advocated for online delivery methods [13,16]. Research has shown that AYA experience challenges in forming new social relationships with non-cancer peers due to their developmental stage and because peers without cancer cannot relate to the problems that accompany a cancer diagnosis [11,14]. However, there remains a paucity of research investigating the specific interpersonal concerns that may discourage AYA from seeking support from cancer peers. Our study has revealed that a significant proportion of AYA (e.g., 50 to 60%) have concerns about being around negative-minded cancer patients, getting close to someone who may die, and hearing emotionally difficult stories. These relevant and important concerns reflect an aversion to engaging in emotion-focused coping and making social comparisons with others who are worse off, both of which can increase distress and lower self-esteem [35,36].

This study has confirmed that AYA are overwhelmingly interested in using a digital app to access peer support. In fact, the desire to connect with a peer navigator doubled if participants had the option through a digital app, where they could browse and select from the profiles of AYA peer navigators that match their criteria. This functionality aligns with the popular profile-based social networking software with which AYA are familiar [16], and offers AYA a degree of choice and control over their interactions with others, which is important in other cancer populations [20]. Research by Panier found that AYA have the desire to use technology to access a navigator, but would prefer to meet in-person for the first interaction [15]. Uniquely, our study, which was conducted before the COVID-19 pandemic, provides evidence in favor of peer support programs for AYA that are exclusively technology-based. This is a particularly important finding given the transition to virtual care necessitated by the COVID-19 pandemic, and will help inform the design and implementation of virtual support programs beyond the pandemic. This study has also re-confirmed [37] that AYA have the desire to give back and help others through the cancer experience. In our cohort, the majority of AYA were interested in volunteering to be a peer navigator, agreed that training for the role would be important, and would be willing to attend an online peer navigation training program. These findings hold promise for the success of a future peer navigation program for AYA, given that training in core competencies is critical for effectiveness and sustainability [38,39].

According to optimal matching theory, the effects of social support will be greatest if matched to the demands of the stressor and the needs of the support seeker [40]. In other words, if emotional support is needed, informational support will be unhelpful. This study indicates a greater need for both emotional (e.g., expression of feelings/concerns, and displays of empathy, caring, and reassurance) and informational support (e.g., resources or advice regarding a problem) from a peer and a peer navigator, rather than companionship (e.g., hanging out and performing normal social things) or practical support (e.g., daily chores, child care, getting to appointments, and help with finances). Other research has found that AYA desire emotional encouragement and information resources from a navigator [15]. In our sample, the desire for companionship and practical support was fairly high, nuanced by the preference for more companionship from a peer, and more practical support from a peer navigator. These findings align with the different types of support typically provided by a peer versus a peer navigator, and demonstrate a need for both. The high proportion of AYA desiring companionship and practical support is expected, given that cancer can disrupt existing relationships with non-cancer peers (11,14) and cause a renewed dependence on parents for practical support and financial assistance [7]. Moreover, practical support could vary based on age, with younger AYA needing assistance with school and basic information regarding health insurance, while older AYA may have concerns regarding their family’s needs and finances [15]. Support from a peer navigator could address these practical concerns, and lower the burden of care for parents and families [18]. 

To our knowledge, this is the first study to have investigated the characteristics that AYA are looking for in a peer navigator. AYA clearly desire to be matched with a peer navigator diagnosed with a similar type of cancer, at a similar age, and who has experienced similar concerns or treatment side effects. Other important matching characteristics included coping style, personality, geographic region, and hobbies. Matching based on sociodemographic factors was least important. The lower importance rankings of education, gender, sexual orientation, and race/ethnicity as matching criteria may be due to the insufficient diversity in the sample, given that most of the participants were college/university educated, and identified as cis-gender, heterosexual, and white or perhaps highlights the inclusivity of this younger demographic. For AYA, a digital app with a matching algorithm may partly address the challenges that AYA experience in finding other cancer patients with whom they can relate. Relatedness is a basic psychological need that is central to effective interpersonal relationships [41]. Further, research has found that patient satisfaction with navigators is largely based on the social and relational skills of the navigator and less on their technical skills [42]. Matching AYA with peer navigators who share similar characteristics could enhance their satisfaction with their navigator [42], and likely, the relevance of the information and support shared [40].

The study has certain limitations. Given its cross-sectional design, there may be some recall bias. While we aimed to obtain a representative sample of AYA both in-person and online to capture AYA across Canada, 80% of participants were from a single region (Ontario). Therefore, AYA living in other regions may have different views, given that AYA peer support programming varies across regions. The study sample is somewhat racially and ethnically diverse, but is not sufficiently diverse in terms of gender, sexual orientation, and rurality. In addition, the data on the rurality of the sample are limited as the response options (urban, suburban, town or rural) were not defined in terms of population density. Finally, there may be a level of selection bias, as those who are interested in peer support likely completed this survey. The degree of selection bias is likely higher among those recruited online versus those approached in clinics, as there was an effort made to approach every eligible patient during the study period. 

## 5. Conclusions

AYA desire support from other cancer peers, even though a significant proportion do not ultimately access peer support. A digital peer navigation program was desired by most and could overcome the barriers that AYA experience in accessing peer support. Future research should consider the specific matching characteristics and multidimensional support needs of AYA in the design of a digital peer navigation program for AYA.

## Figures and Tables

**Table 1:** Participant characteristics.

Characteristic	Category	Count (%), Unless Otherwise Specified
Age (years), mean (SD), (*n* = 419)		31.3 (6.3)
Age at Diagnosis (years), mean (SD), (*n* = 412)		28.0 (6.8)
Time Since Diagnosis (years), mean (SD), (*n* = 415)		3.3 (3.8)
Cancer Type (*n* = 419)	Breast	91 (21.7)
	Hodgkin’s Lymphoma	47 (11.2)
	Testicular	41 (9.8)
	Leukemia	36 (8.6)
	Sarcoma	35 (8.4)
	Thyroid	32 (7.6)
	Gastrointestinal, colorectal, liver	31 (7.4)
	Gynecological (Cervical, ovarian, uterine)	27 (6.4)
	Non-Hodgkin’s Lymphoma	25 (6.0)
	Other	54 (12.9)
Treatment Types (*n* = 419)	Chemotherapy	311 (74.2)
	Hormone	70 (16.7)
	Radiation	175 (41.8)
	Surgery	254 (60.6)
	Bone Marrow Transplant	22 (5.3)
	Other	51 (12.2)
Gender (*n* = 337)	Woman	218 (65.1)
	Man	117 (34.7)
	Transgender	0 (0)
	Prefer not to answer	2 (0.6)
Sexual Orientation (*n* = 335)	Heterosexual	286 (85.4)
	Homosexual/Bisexual/Other	41 (12.3)
	Prefer not to answer	8 (2.4)
Country of Birth (*n* = 337)	Canada	251 (74.5)
	Other	86 (25.5)
Race/Ethnicity (*n* = 336)	White	212 (63.1)
	Not White/Not Indigenous	111 (34.5)
	Indigenous	13 (3.9)
Province where you currently live (*n* = 337)	Newfoundland	6 (1.8)
	Nova Scotia	6 (1.8)
	Quebec	11 (3.3)
	Ontario	274 (81.3)
	Manitoba	11 (4.2)
	Saskatchewan	1 (0.3)
	Alberta	13 (2.7)
	British Columbia	9 (2.7)
	Northwest Territories	1 (0.3)
	I do not currently live in Canada	2 (0.6)
Size of setting where you currently live (*n* = 336)	Urban or suburban (e.g., city)	292 (86.9)
	Town or rural (e.g., country)	44 (13.1)
Current school or employment status (*n* = 338)	Employed and/or Student	272 (80.5)
	Unemployed	66 (19.5)
Highest level of education (*n* = 340)	Completed/Some University *	216 (63.5)
	Completed/Some College *	84 (23.7)
	High school or less	28 (8.9)
Personal income (*n* = 336)	Less than CAD 40,000 or no income/ not working	161 (47.9)
	CAD 40,000 to less than CAD 80,000	87 (25.9)
	CAD 80,000 or more	49 (14.6)
	Prefer not to answer	38 (11.3)

* In Canada, colleges offer Diploma and Certificate programs, while universities offer Bachelor, Master’s, and Doctoral degrees.

**Table 2:** Needs and preferences for peer support.

Characteristic (*n*)	Category	Count (%)
Desire to connect with other AYA (*n* = 380)	Yes	291 (76.6)
Talked to a medical professional regarding peer support (*n* = 382)	Yes, a medical professional initiated the conversion	191 (50.0)
	Yes, I initiated the conversation	46 (12.0)
	No	145 (38.0)
Received a referral to a peer support program (*n* = 383)	Yes	143 (37.3)
Methods used to connect with other AYA (*n* = 379)	Social media	152 (40.1)
	Cancer organizations	117 (30.9)
	In-person support groups	85 (22.4)
	Camps, retreats, and adventure groups	67 (17.7)
	Online discussion forums	51 (13.5)
	Healthcare professional	50 (13.2)
	Conference	50 (13.2)
	Telephone support service	21 (5.5)
	Digital apps	12 (3.2)
Can you count on another AYA to (fill in with your answer)		
Provide you with good information or advice regarding a problem? (*n* = 372)	I would like/or I would like more of this type of peer support	159 (42.7)
	I have this type of support	95 (25.5)
	Not needed	118 (31.7)
Provide you with emotional support? (*n* = 372)	I would like/or I would like more of this type of peer support	168 (45.2)
	I have this type of support	85 (22.8)
	Not needed	119 (32.0)
Provide you with practical help with things, such as daily chores, child care or getting to appointments? (*n* = 368)	I would like/or I would like more of this type of peer support	105 (28.5)
	I have this type of peer support	22 (6.0)
	Not needed	241 (65.5)
Hang out with and perform normal social things? (*n* = 370)	I would like/or I would like more of this type of peer support	161 (43.6)
	I have this type of peer support	71 (19.2)
	Not needed	138 (37.3)
Satisfaction with peer support received from other AYA (*n* = 379)	Very Satisfied/Satisfied	139 (36.7)
	Neutral	49 (12.9)
	Very Dissatisfied/Dissatisfied	10 (2.6)
	N/A, I have not received support from other AYA	181 (47.8)

**Table 3:** Barriers to accessing peer support.

Frequency	Variable (*n*)	Count (%)
1	In-person support programs were not convenient to attend (*n* = 373)	284 (76.1)
2	Finding another AYA who I can relate to was difficult for me (*n* = 370)	268 (72.4)
3	AYA specific in-person support programs were hard to find (*n* = 371)	261 (70.4)
4	Unsure of where or how to find other AYA (*n* = 373)	257 (68.9)
5	Uncomfortable attending in-person support programs (*n* = 373)	247 (66.12)
6	Concerned about presence around negative minded cancer patients (*n* = 372)	223 (59.9)
7	Concerned about getting close to someone who might die (*n* = 370)	214 (57.8)
8	Concerned about hearing emotionally difficult stories (*n* = 371)	208 (56.1)
9	Discussing my health condition on social media worries me due to privacy (*n* = 373)	202 (54.2)
10	Desire to reconnect with healthy peers and not other cancer patients (*n* = 368)	146 (39.7)
11	Using social media is difficult for me (*n* = 373)	31 (8.3)
12	Access to a computer or mobile device is difficult for me (*n* = 372)	24 (6.5)
13	Using a computer or mobile device is difficult for me (*n* = 373)	15 (4.0)

Participants were asked to rate the extent to which these variables were barriers on a 4-point Likert scale (1 = no problem, 2 = not sure, 3 = somewhat of a problem, 4 = big problem). Responses were dichotomized as no barrier vs. not sure, somewhat, big barrier.

**Table 4:** Interest and preferences for digital peer navigation.

Variable (*n*)	Category	Count (%)
Interest in being connected with a peer navigator (*n* = 380)	Yes	186 (48.9)
If no: (*n* = 195)		
	I do not think I need support	82 (42.1)
	I do not like to talk about my problems	21 (10.8)
	I believe I have adequate support	88 (45.1)
	Other	27 (13.8)
Interest in being a peer navigator (*n* = 367)	Yes	231 (62.9)
Importance of training for the peer navigator role? (*n* = 379)	Yes (slight to very important)	257 (67.8)
	No	86 (22.7)
	Unsure	36 (9.5)
Willingness to attend an online peer navigator training program (*n* = 366)	Yes	231 (63.1)
Interest in using a digital app to connect with a peer navigator (*n* = 378)	Yes	310 (82.0)
Digital app communication methods (*n* = 358)	One-on-one	259 (72.3)
	In a group	242 (67.6)
Types of support desired from a peer navigator? (*n* = 436)	Emotional support	329 (90.1)
	Information support	326 (89.1)
	Social companionship	284 (78.0)
	Practical support	269 (73.6)
Best time to connect with a peer navigator (*n* = 368)	During diagnosis	250 (67.9)
	Before treatment	248 (67.4)
	During treatment	295 (80.2)
	After treatment	256 (69.6)
	If cancer recurs or spreads	253 (68.8)

**Table 5:** Peer matching characteristics.

Rank	Matching Characteristics (*n*) *	Count (%)
1	Type of cancer (*n* = 372)	329 (88.4)
2	Specific concerns (e.g., side effects, return to work) (*n* = 368)	317 (86.1)
3	Age at diagnosis (*n* = 367)	316 (86.1)
4	Treatments received/considering (*n* = 369)	314 (85.1)
5	Current age (*n* = 368)	302 (82.1)
6	Stage of disease (*n* = 367)	278 (75.7)
7	Coping style (*n* = 367)	268 (73.0)
8	Personality style (*n* = 368)	237 (64.4)
9	Geographic region where you live (*n* = 365)	229 (62.7)
10	Hobbies/interests (*n* = 364)	191 (52.5)
11	Gender (*n* = 367)	167 (45.5)
12	Hospital where you were treated (*n* = 368)	144 (39.1)
13	Religion/spirituality (*n* = 357)	132 (37.0)
14	Relationship status (e.g., single, married) (*n* = 367)	122 (33.2)
15	Education (*n* = 368)	115 (31.3)
16	Sexual orientation (*n* = 367)	63 (17.2)
17	Race/ethnicity (*n* = 368)	29 (7.9)

* Participants were asked to rate the extent to which these variables were important matching characteristics on a 4-point Likert scale (1 = not at all important, 2 = not sure, 3 = somewhat important, 4 = very important). Responses were dichotomized as not important vs. not sure, somewhat important, very important.

## Supplementary Materials

The following supporting information can be downloaded at: https://www.mdpi.com/article/10.3390/curroncol29020099/s1, Survey of Peer Support Needs and Preferences for Digital Peer Navigation among of Adolescents and Young Adults with Cancer.
