# Supplementary material for: Peer Support Needs and Preferences for Digital Peer Navigation among Adolescent and Young Adults with Cancer: A Canadian Cross-Sectional Survey
Source: Curr Oncol. 2022 Feb 16;29(2):1163–75. doi: 10.3390/curroncol29020099 (PMC8870696; doi:10.3390/curroncol29020099)
Supplement: Supplementary file 1 [file curroncol-29-00099-s001.zip › curroncol-1571938-supplementary.pdf]

Supplementary Material: Survey of Peer Support Needs and Preferences for Digital Peer Navigation among of Adolescents and Young Adults with Cancer

Eligibility Questions

Please answer the following questions to confirm your eligibility for this study:

1. **Were you diagnosed with cancer between 15 and 39 years of age?**
   0☐ No > *Unfortunately, you are not eligible to participate in this survey.*
   1☐ Yes

2. **Have you received cancer treatment within the last 10 years?**
   0☐ No > *Unfortunately, you are not eligible to participate in this survey*.
   1☐ Yes

Part A: Cancer Specific Background Information About You

A1. Today's Date: ________/_________/_________
Day Month Year

A2. What is your age? ________ years

A3. When were you first diagnosed with cancer: __________________/_________
Day Month

A4. What type of cancer were you diagnosed with?
1☐ Breast Cancer
2☐ Cervical Cancer
3☐ Colorectal Cancer
4☐ Hodgkin's Lymphoma
5☐ non-Hodgkin Lymphoma
6☐ Leukemia
7☐ Melanoma
8☐ Testicular Cancer
9☐ Thyroid Cancer
10☐ I don't know
11☐ Other. Please specify: _____________________

A5. What stage was your cancer when you were first diagnosed?
1☐ Stage 1
2☐ Stage 2
3☐ Stage 3
4☐ Stage 4
5☐ I don't know

A6. Have you had a cancer recurrence?
1☐ Yes
0☐ No

A7. Have you been diagnosed with any other types of cancer? If yes, please describe date of diagnosis and type of cancer:

Type of Cancer	Month	Year

A8. Are you living with metastatic cancer?

1☐ Yes
0☐ No

A9. Are you currently undergoing treatment?

1☐ Yes
0☐ No

A10. Which types of cancer treatments have you undergone or are you currently receiving? Check all that apply.

1☐ Drug or Chemotherapy
2☐ Hormone Therapy
3☐ Radiation Therapy
4☐ Surgery
5☐ Bone marrow or stem cell transplant
6☐ Other, please specify: ____
7☐ None.

A11. To the best of your knowledge, are you now cancer free?

1☐ Yes
0☐ No
2☐ I don't know

A12. Based on your interactions with your doctors, nurses, and other health care professionals, how would you rate the quality of care you have received since your cancer diagnosis?

1☐ Poor 2☐ Fair 3☐ Good 4☐ Very good 5☐ Excellent

Part B: Preferences for Peer Support from other Adolescents and Young Adults (AYA) Diagnosed with Cancer

B1. Since your cancer diagnosis, have you ever wanted to connect (e.g. meet in person, talk on the phone or online) with other **adolescents and young adults with cancer** **(AYA)**? This includes people who were diagnosed with cancer between the ages of 15 and 39.

1☐ Yes
0☐ No

B2. Since your diagnosis, has a medical professional (e.g. oncologist, nurse, psychologist) talked to you about peer support? Peer support is information, emotional or practical support from another person who has experienced cancer.

1☐ Yes, a medical professional initiated the conversation
2☐ Yes, I initiated the conversation
0☐ No

B3. Since your cancer diagnosis, have you ever received a referral to a peer support program (e.g. peer support group, Young Adult Cancer Canada) from a medical professional?

1☐ Yes
0☐ No

B4. Since your diagnosis, have you connected with other AYA through any of the following sources? Select all that apply.

1☐ My health care professional
2☐ Telephone peer support call service
3☐ In-person support group
4☐ Camps, retreats, and adventure programs
5☐ Conference
6☐ Cancer organizations (e.g. Young Adult Cancer Canada -YACC, Cancer Fight Club, Stupid Cancer)
7☐ Social Media (E.g. Facebook, Twitter, Instagram, Snapchat etc.)
8☐ Online discussion forum
9☐ Digital apps that connect you with other AYA (e.g. Stupid Cancer, Upopolis)
10☐ Other, please specify: ____
11☐ I did not try to connect with other AYA

B5. Now we would like to know some of the factors that might have made it difficult for you to connect with other AYA, whether or not you tried to connect with other AYA. **Please select the BEST response.**

**Factor**	**No problem**	**Somewhat of a problem**	**Big problem**	**Not sure**
a. Being unsure of where or how to find other AYA	1☐	2☐	3☐	4☐
b. Being uncomfortable attending in-person support programs	1☐	2☐	3☐	4☐
c. In-person support programs were not convenient to attend	1☐	2☐	3☐	4☐
d. AYA specific in-person support programs were hard to find	1☐	2☐	3☐	4☐

Factor	No problem	Somewhat of a problem	Big problem	Not sure
e. Access to a computer or mobile device was not available	1☐	2☐	3☐	4☐
f. Using a computer or mobile device is difficult for me	1☐	2☐	3☐	4☐
g. Using social media is difficult for me	1☐	2☐	3☐	4☐
h. Discussing my health condition on social media worries me because of privacy concerns	1☐	2☐	3☐	4☐
i. Finding another AYA who I can relate to was difficult for me	1☐	2☐	3☐	4☐
j. Being concerned about hearing emotionally difficult stories	1☐	2☐	3☐	4☐
k. Being concerned about being around negative minded cancer patients	1☐	2☐	3☐	4☐
l. Being concerned about getting close to someone who might die	1☐	2☐	3☐	4☐
m. Wanting to reconnect with healthy peers and not other cancer patients	1☐	2☐	3☐	4☐
n. Other. Please specify: ______________________________				
I <u>did not</u> try to connect with other AYA.	Yes ☐			

B6. Now we would like to know about the **type of peer support from other AYA** that is available to you or that you may want. **Check the BEST response.**

	No, not needed	No, but I would like this type of peer support	Yes, I currently have this type of support	Yes, and I would like <u>more</u> of this type of peer support
a. Can you count on another AYA to provide you good information or advice about a problem?	1☐	2☐	3☐	4☐
b. Can you count on another AYA to provide you with emotional support (talking over problems or helping you to make a difficult decisions).	1☐	2☐	3☐	4☐
c. Can you count on another AYA to help you do practical things like daily chores, child care or getting to appointments.	1☐	2☐	3☐	4☐
d. Can you count on another AYA to hang out with and do normal social things?	1☐	2☐	3☐	4☐

B7. In general, how satisfied are you with the peer support that you have received from other AYA so far?

1☐ Very dissatisfied
2☐ Dissatisfied
3☐ Neutral

4☐ Satisfied
5☐ Very satisfied
6☐ Not applicable. I have not received support from other AYA.

B8. Would you be interested in being connected with a **peer cancer survivor**? A peer cancer survivor is an AYA who has been through the cancer experience.

1☐ Yes
0☐ No. If no, what are the reasons why not?
  a☐ I don't think I need support
  c☐ I don't like to talk about my problems
  d☐ I believe I have adequate support
  e☐ Other, please specify: ____

B9. Would you be interested in being connected with a **peer navigator**? A peer navigator is a cancer survivor who has been **trained** to help others through the cancer experience by providing informational, emotional and practical support.

1☐ Yes
0☐ No. If no, what are the reasons why not?
  a☐ I don't think I need support
  c☐ I don't like to talk about my problems
  d☐ I believe I have adequate support
  e☐ Other, please specify: ____

B10. How important is it for you to be connected with a peer cancer survivor or navigator who has **received training** to support others through the cancer experience?

1☐ Not important
2☐ Slightly important
3☐ Moderately important
4☐ Important
5☐ Very important
6☐ Not sure

B11. How would you want to **communicate** with a peer cancer survivor or navigator? Check all that apply.

1☐ In-person
2☐ By telephone
3☐ By texting
4☐ Through email
5☐ On social media (Facebook, Twitter, Instagram)
6☐ Through video (e.g. Skype, Facetime etc.)
7☐ Other. Please describe:____

B12. Would you be interested in using a **digital app** to connect and communicate with a peer cancer survivor or navigator? You could view the profiles of AYA that match your selection criteria (e.g. age, cancer diagnosis) and choose the AYA who you would like to talk to.

1☐ Yes
0☐ No. If no, what are the reasons why not?____

B13. In this digital app would you want to be able to communicate. **Check all that apply:**

1☐ One-on-one with one peer cancer survivor or navigator
2☐ One-on-one with one or more peer cancer survivors or navigators
3☐ In a group with many other AYA, like an online discussion forum
4☐ Other. Please describe:____

B14. How important would it be for you to be **matched** with a peer cancer survivor or navigator who is similar to you in terms of the following characteristics? **Check the BEST response for each row.**

	Not important at all	Somewhat important	Very Important	Not sure
a. Age at diagnosis	1☐	2☐	3☐	4☐
b. Current age	1☐	2☐	3☐	4☐
c. Type of cancer	1☐	2☐	3☐	4☐
d. Stage of disease (e.g. 1, 2, 3, 4)	1☐	2☐	3☐	4☐
e. Treatments received/considering	1☐	2☐	3☐	4☐
f. Specific concerns (e.g. side effects, return to school/work)	1☐	2☐	3☐	4☐
g. Hospital where you were treated	1☐	2☐	3☐	4☐
g. Geographic region where you live	1☐	2☐	3☐	4☐
h. Gender	1☐	2☐	3☐	4☐
i. Sexual orientation	1☐	2☐	3☐	4☐
j. Race/Ethnicity	1☐	2☐	3☐	4☐
k. Relationship Status (e.g. single, married)	1☐	2☐	3☐	4☐
l. Education	1☐	2☐	3☐	4☐
m. Personality style	1☐	2☐	3☐	4☐
n. Coping style	1☐	2☐	3☐	4☐
o. Hobbies/interests	1☐	2☐	3☐	4☐
p. Religion/spirituality	1☐	2☐	3☐	4☐
n. Other. Please specify:____				

B15. **When** would it be most helpful to be connected with a peer cancer survivor or navigator? Check all that apply.
1☐ During diagnosis

2☐ Before treatment
3☐ During treatment
4☐ After treatment
5☐ If cancer recurs or spreads
6☐ Other. Please specify:____

B16. Now, we would like to know what type of support you would like to receive from a peer cancer survivor or navigator. Please rate the importance of each of the following types of support from being very important to not important at all.

	Not important at all	Slightly important	Moderately important	Important	Very important
a. **Informational support** (e.g. Information about cancer, its treatment, side effects, AYA-specific resources, hospital programming, transition to survivorship)	1☐	2☐	3☐	4☐	5☐
b. **Emotional support** (e.g. Discussion of experiences, validation of feelings and concerns, and coping with the cancer and its treatment and its impact on your life, your relationships and your plans etc.)	1☐	2☐	3☐	4☐	5☐
c. **Practical support** (e.g. Assistance to attend appointments, arrange leave/return to school or work, financial support, childcare, travelling etc.)	1☐	2☐	3☐	4☐	5☐
d. **Social companionship** (e.g. Someone to hang out with and do normal social things)	1☐	2☐	3☐	4☐	5☐
e. Other. Please specify:____					

B17. Would you be interested in volunteering to be a peer navigator to support other AYA through the cancer journey? (You would be provided training and professional support to be a peer navigator. You could communicate with your matches online, by phone or in-person.)

The primary role of a Peer Navigator is to:

- Assist patients to identify needs and overcome barriers to getting their needs met
- Empower patients by working with them to identify their own strengths, abilities and coping strategies
- Discuss experiences, validate feelings and concerns

1☐ Yes
0☐ No. If no, what are the reasons why not:____

B18. Have you ever <u>provided peer support</u> to other people affected by cancer?
1☐ Yes
0☐ No

B19. Have you received training to provide peer support to other people affected by cancer?
1☐ Yes. If yes, please describe:____ ___
0☐ No

B20. Would you be willing to attend a peer navigator training course to become a peer navigator?

This course would provide you with the knowledge, skills and competencies to be a peer navigator for other AYA. It would be delivered online over the course of 6 weeks, with 1 to 2 in-person workshops. It would require a commitment of about 4 hours per week, or 24 hours in total. You would be provided a certificate at the completion of the course.

1☐ Yes
0☐ No. If No, please explain why not:____

Part C: Overall Health and Wellbeing

[This section contains validated measures. These measures were not reported in this manuscript. Please contact the corresponding author for more information]

Part D: Background Information About You

In this final section, we are interested in some background information about you. We want to ensure that people with a range of backgrounds are involved in this study.

D1. What is the highest level of education that you have completed?
1☐ Elementary school
2☐ Some high school
3☐ Completed high school
4☐ Some college or vocational school
5☐ Completed college or vocational school
6☐ Some university
7☐ Completed university
8☐ Other. Please specify:_____________________________

D2. What is your current school or employment status? Please select all that apply.
1☐ Part-time student
2☐ Full-time student
3☐ Working part-time
4☐ Working full-time
5☐ Unemployed and looking for work
6☐ Unemployed and not looking for work
7☐ Full-time homemaker or family caregiver
8☐ Other. Please specify:____________________

D3. Has your school/employment status changed because of your cancer or its treatment? Please select all that apply.
1☐ It has not changed because of my cancer or its treatment
2☐ I was unable to work and went on disability benefits.
3☐ I quit working completely
4☐ I quit going to school completely
5☐ I changed my work status from full-time to part-time
6☐ I changed my school status from full-time to part-time
7☐ I took more than 2 weeks total time off from work

8☐ I took more than 2 weeks total time off from school
9☐ I asked for work flexibility (e.g. worked remotely, changed my hours)
10☐ I closed my business
11☐ Other. Please specify: ______________________

D4. Do you currently live alone or with others?
1☐ Live alone
2☐ Live with others (e.g. parent, roommate, spouse, partner, brother, sister, children)

D5. Are you now responsible for raising any children under the age of 18?
1☐ Yes
0☐ No

D6. What is your sex?
1☐ Male
2☐ Female
3☐ Prefer not to answer

D7. What is your gender?
1☐ Male
2☐ Female
3☐ TransMale/Transman
4☐ TransFemale/Transwoman
5☐ Genderqueer
6☐ Other. Please specify:_______________________
7☐ Prefer not to answer

D8. How would you describe your sexuality?
1☐ Heterosexual
2☐ Homosexual
3☐ Bisexual
4☐ Prefer not to answer
5☐ Other (Please specify:_______________________

D9. Were you born in Canada?
1☐ Yes
2☐ No. If no, in which country were you born?____________________

D10. People in Canada come from many different backgrounds. Are you: (check all that apply)
1☐ Indigenous (e.g. First Nations, Metis, Inuit)
2☐ White
3☐ South Asian (e.g. Indian, Pakistani, Sri Lankan…)
4☐ Chinese
5☐ Black
6☐ Filipino
7☐ Latin American
8☐ Arab
9☐ Southeast Asian (e.g. Vietnamese, Cambodian, Malaysian, Laotian,)

10☐ West Asian (e.g. Iranian, Afghan)
11☐ Korean
12☐ Japanese
13☐ African
14☐ Caribbean
16☐ Other. Please specify:____________________

D11. What language do you speak most often at home?

1☐ English
2☐ French
3☐ Other. Please specify: ______________________

D12. In which province or territory do you live?

1☐ Newfoundland and Labrador
2☐ Prince Edward Island
3☐ Nova Scotia
4☐ New Brunswick
5☐ Quebec
6☐ Ontario
7☐ Manitoba
8☐ Saskatchewan
9☐ Alberta
10☐ British Columbia
11☐ Yukon
12☐ Northwest Territories
13☐ Nunavut
14☐ I do not live in Canada

D13. In what type of setting do you live most of the year?

1☐ Urban or Suburban (city)
2☐ Town or Rural (country)

D14. Which of the following categories best describes your personal income in 2017?

1☐ Less than $20,000
2☐ $20,000 to less than $40,000
3☐ $40,000 to less than $60,000
4☐ $60,000 to less than $80,000
5☐ $80,000 or more
6☐ No income. (I am not working)
7☐ Prefer not to answer

Thank you for completing this survey.

If you are interested in participating in a prize draw, receiving the results, or participating in a workshop to help us design a digital app please turn the page!
